# Patan hospital experience in treating philadelphia chromosome/BCR-ABL1 positive chronic myeloid leukemia patients with gleevec (imatinib mesylate); the first generation specific tyrosine kinase inhibitor

**DOI:** 10.1186/1471-2326-10-8

**Published:** 2010-12-07

**Authors:** Gyan K Kayastha, Padma Gurung, Paras K Acharya, Buddhi P Paudyal, Bruce Hayes, Mark Zimmerman, Arjun Karki, Aaron S Mansfield

**Affiliations:** 1 Department of Internal Medicine, Patan Hospital, Lalitpur Nepal; 2 Department of Emergency Medicine, Patan Hospital, Lalitpur, Nepal; 3 Nick Simon Institute, Lalitpur, Nepal; 4 Division of Hematology of the Department of Internal Medicine, and Department of Oncology, Mayo Clinic, Rochester, MN, USA

## Abstract

**Background:**

Chronic Myeloid Leukemia (CML) is caused by the abnormal fusion protein BCR-ABL1, a constitutively active tyrosine kinase and product of the Philadelphia chromosome. Gleevec (Imatinib mesylate) is a selective inhibitor of this kinase. Treatment with this agent is known to result in hematologic, cytogenetic, and molecular responses. Patan hospital (Patan, Nepal) is one of the Gleevec International Patient Assistance Program (GIPAP) centers for patients with CML.

**Methods:**

A total of 106 Philadelphia positive CML patients were enrolled in our center between Feb 2003 and Jun 2008, and 103 of them were eligible for cytogenetic and/or hematologic response analyses.

**Results:**

Out of 103 patients, 27% patients underwent cytogenetic analysis. Imatinib induced major cytogenetic responses in 89% and complete hematologic responses in almost 100% of the patients with confirmed CML. After a mean follow up of 27 months, an estimated 90% of the patients on imatinib remained in hematologic remission and more than 90% of the patients are still alive. About 30% of patients developed some form of manageable myelosuppression. A few patients developed non-hematologic toxic side effects such as edema and hepatotoxicity.

**Conclusions:**

Our study demonstrates that imatinib is safe to use in a developing country. Furthermore, we demonstrate that imatinib is very effective and induced long lasting responses in a high proportion of patients with Ph chromosome/BCR-ABL1 positive CML. Imatinib is well tolerated by our patients. The lack of cytogenetic analysis in the majority of our patients hindered our ability to detect inadequate responses to imatinib and adjust therapy appropriately.

## Background

Chronic myeloid leukemia (CML), is a myeloproliferative disorder caused by a translocation between chromosomes 9 and 22 that results in the Philadelphia (Ph) chromosome [t(9;22) (q34;q11)] [1]. The Ph chromosome was found to encode a reconstituted fusion gene known as *BCR-ABL1*, which is the principal cause of CML[2]. The *BCR-ABL1 *fusion gene encodes the BCR-ABL1 oncoprotein. This oncoprotein, which is constitutively active due to its uncontrolled tyrosine kinase activity, activates signal transduction pathways that lead to DNA replication and cell proliferation[3-5].

The incidence of CML is similar in countries around the world and may affect ethnic groups equally. The annual incidence of CML in the US is 1.6 cases per 100,000 adults (approximately 5000 new cases per year), with a male-to-female ratio of 1.4 to 1[6,7]. The median age at diagnosis is approximately 55 years, with fewer than 10% of patients under the age of 20 years[8]. During the chronic phase of the disease there is clonal expansion of myeloid cells which continue to differentiate. Over time, however, the leukemic clone loses their ability to differentiate, and the disease ultimately progresses to blast crisis[3,9,10].

Until the 1980s, CML was regarded as incurable and thus inevitably fatal. Only some selected patients could afford treatment with, and in many cases were cured by allogeneic stem cell transplantation. However, because of the scarcity of matching donors and the high incidence of potentially fatal graft-versus-host disease in older patients, allogeneic stem cell transplantation is often not a feasible treatment strategy. Imatinib[11-16], is a potent first generation selective inhibitor of tyrosine kinase activity of BCR-ABL1. It was first approved by the Food and Drug Administration for CML in May 2001 and has been proven to be one of the most effective medications in achieving hematologic and cytogenetic remissions. Imatinib is a major break-through in the management of CML and has become the standard first line treatment strategy in the management of CML[17].

Patan hospital is the first Gleevec International Patient Assistance Program (GIPAP) center in Nepal. GIPAP was introduced by Dr. Zimmerman, the medical director of this hospital at that time, for patients with Ph chromosome/BCR-ABL1 positive CML since February 2003. Under this program patients with Philadelphia chromosome/BCR-ABL1 positive CML who cannot afford Imatinib receive it free of cost.

## Methods

### Study Design

Patients with Ph Chromosome/BCR-ABL1 positive CML at any stage of the disease were eligible for enrolment in GIPAP irrespective of prior therapy. Patients diagnosed with CML by blood and by bone marrow evaluation in our hospital, or referred from other hospitals, were sent for Ph chromosome/BCR-ABL1 analysis. All positive cases were enrolled in GIPAP. Patients who were diagnosed with CML and found to be Ph chromosome/BCR-ABL1 positive in other hospitals and referred to our hospital were also enrolled in GIPAP. Hydroxycarbamide and other medications for the treatment of CML were tapered off over a few days. Before commencement of the treatment with imatinib patients were screened for adequate renal and hepatic function, along with assessment of their performance status. Written informed consent was obtained from all patients before they were enrolled in GIPAP. Ethical approval was not considered necessary as GIPAP made available treatment that was already considered standard of care.

Patients were assigned to receive imatinib 400 mg/day for chronic phase and 600 mg/day for accelerated phase and blast crisis. Patients received continuous therapy unless unacceptable adverse effects or disease progression occurred. No other anticancer therapy was given. All patients received allopurinol until the total WBC count was 20,000/mL or less.

Complete blood counts and liver function tests were obtained once a week for the first month, every two weeks for next 2 months, and every 6 weeks afterwards. Patients were monitored for any adverse effects, clinical, and hematologic responses. Cytogenetic analyses were done for those patients who could afford this test, and patients who developed resistance to imatinib underwent mutation analysis. Only three patients underwent mutation analysis. Standard protocol for managing adverse effects during imatinib therapy was followed.

### Assessment of Toxicity and Response

Safety assessments included the evaluation of adverse events and vital signs, hematological tests, biochemical tests, and physical examination. Toxicity was graded in accordance with the Common Toxicity Criteria of the National Cancer Institute.

## Results

### Patient Enrolment and Characteristics

Enrolment in GIPAP was started in Feb 2003. During the period from February 2003 to June 2008, a total of 106 patients ranging from age 10 to 71 years (median 39) were enrolled in GIPAP in our hospital. Starting with the first patient in February 2003, the enrolment steadily increased over the years. The most patients were enrolled in 2006 and 2007, with 26 and 23 patients in each of those years respectively. Most of the patients were from the Kathmandu and Lalitpur districts. There were patients from 37 of the 75 districts of Nepal. There were 70 males and 36 female patients (Table 1).

**Table 1:** Patient characteristics

Patient categories	Number of Patients	Eligible Patients		
		HR	CR	Total
Registered in GIPAP	*106*	74	29	103
Diagnosed in Patan	*29*	16	12	28
Diagnosed at Other Hospitals	*77*	58	17	75
Excluded	*3*	0	0	0
Chronic Phase	98	69	27	96
Accelerated Phase	5	5	0	5
Blast Crisis	3	0	2	2
Male/Female	70/36	51/23	17/12	68/35
Follow up (Mean/Median) (m)	27/24	23/19	39/42	27/24
Age (mean/median)	39/39	39/40	38/38	39/39
Lost to f/u	8	8	0	8
Mortality	2	2	0	2

HR = hematologic response

CR = cytogenetic response

The majority of the patients were in the 31-50 years age group (51%) (Table 2). Of the total 106 patients, 29 patients were diagnosed at Patan Hospital and the rest (77 patients) were referred from other hospitals for GIPAP assistance. Of the 77 referred patients 36 patients were diagnosed by blood and/or bone marrow in different hospitals in Nepal and required additional investigations including Ph chromosome/BCR-ABL1 translocation status. The remaining 41 patients were referred from other hospitals for GIPAP enrolment after confirmation with Ph chromosome/BCR-ABL1 translocation analysis.

**Table 2:** GIPAP enrolment by age groups

GIPAP Enrollment by Age Groups	
Age groups	No of patients
< 20	13
21-30	16
31-40	31
41-50	23
51-60	13
61-70	8
71-80	2
Total	106

Of the total 106 patients, three patients were excluded from the study and the remaining 103 patients were eligible for analysis. The three patients excluded were: one who died before starting imatinib, one who registered but did not show up afterwards, and one who registered but then moved to another country. Among the 28 patients who were diagnosed at Patan hospital, only 10 patients presented with abdominal complaints such as abdominal fullness, swelling, mass, early satiety, abdominal discomfort or pain. The remaining patients presented with other diverse complaints.

Of the 103 patients, 96 patients were in chronic phase, 5 patients were in accelerated phase, and two patients were in blast crisis at the time of presentation. During enrolment, there were 61 patients on hydroxycarbamide, one on busulfan, and one on imatinib. Two other patients were taking combination therapy; one with hydroxycarbamide and imatinib and the other one was on hydroxycarbamide with busulfan (Table 3). The mean follow up was 27 months (median 24, range 1-66). Twenty-nine patients had repeated quantitative Ph chromosome/BCR-ABL1 status; most of them by Fluorescent In-Situ Hybridization (FISH) method.

**Table 3:** Medication use at enrolment

Medication Use at Presentation	
Medications	No
Hydroxycarbamide	61
Busulfan	1
Imatinib	1
Hydroxycarbamide+Imatinib	1
Hydroxycarbamide+Busulfan	1
No anticancer drugs	41
	
Total	106

### Safety profile

Imatinib was generally well tolerated. The most notable adverse effects were some form of myelosuppression: neutropenia and/or thrombocytopenia. Grade 1 or 2 elevation of liver enzymes, edema and weight gain, rashes, nausea, vomiting and diarrhea were other common adverse effects. Patients treated with higher doses of imatinib were more likely to develop grade 1 or 2 nausea, edema, or diarrhea than patients given lower doses of the drug. Neutropenia and/or thrombocytopenia were the most frequent adverse effects and occurred in about 30% of the patients (Table 4).

**Table 4:** Adverse effects

Side Effects	No of Patients	Side Effects	No of Patients
**Wt Gain**	6	Pigmentation	4
**Rashes**	6	**Hepatotoxicity**	**9**
**Swelling**	9	**Myelosuppresion**	**31**
Nausea	5	Gum bleeding	3
Vomiting	5	Mild arthralgia	2
Diarrhea	5	Others	9

There was a total of 2 deaths and 8 patients lost to follow up. Of the 2 deaths both of them died due to disease progression and subsequent complications. Of the 8 patients lost to follow up, two patients who did not return for follow-up were found to have died due to disease progression and blast crisis. The causes of death of the other 6 patients are not known.

### Hematologic and cytogenetic responses

Out of 103 patients, 15% of the patients were already in complete hematologic remission before enrolment in GIPAP. Seventy-eight percent of the patients went into complete hematologic remission within 3 months of starting imatinib and only seven percent of the patients took more than 3 months for complete hematologic remission. Of the 29 patients who had cytogenetic analysis done, 26 (89.66%) patients had major cytogenetic response [20 (68.97%) Complete Response and 6 (20.69%) Partial Response], 2 (6.89%) patients had minor cytogenetic responses, and 1 (3.45%) patient had minimal cytogenetic response (Table 5). The cytogenetic testing was performed between 9 and 61 months (median 26 months) from enrolment in patients who were able to afford testing.

**Table 5:** Hematologic and cytogenetic analyses

Hematologic Analysis			Cytogenetic Analysis		
Total patients	106		Complete	20	68.97%
Excluded	3		Partial	6	20.69%
Total Patients Eligible	103	100%	Major	26	89.66%
CHR at Presentation	15	15%	Minor	2	6.89%
CHR in <3 months	81	78%	Minimal	1	3.45%
CHR in >3 months	7	7%	Total	29	

CHR: complete hematologic response

Of the 29 patients who underwent cytogenetic analysis, four patients who had had major cytogenetic responses initially showed minimal to minor responses with subsequent FISH analysis. Two of these patients were actually taking reduced doses of imatinib. Another patient temporarily stopped taking imatinib. All three of them responded to dose escalation. The fourth patient responded temporarily to an increased dose but eventually relapsed. This patient who relapsed had been to other hospitals in India and had been treated with interferon and other chemotherapeutic medications before starting imatinib. He had a complete hematologic response only for few months after the beginning of therapy at our hospital. He underwent dose escalation to 600 mg/day initially and then to 800 mg/day, but complete hematologic response was not achieved. Quantitative BCR/ABL by FISH repeated after 6 months showed partial cytogenetic response, but repeat analysis showed lack of response. Subsequent mutation analysis revealed a F359V mutation in the ABL domain of the BCR/ABL oncogene. This patient may have developed secondary resistance due to the mutation. Even with the change of medication to the second generation tyrosine kinase inhibitor nilotinib, his response was not satisfactory. While preparing for this presentation this patient started taking dasatinib and he is responding well to this drug. One more patient who developed secondary hematologic failure showed loss of complete cytogenetic response upon repeat cytogenetic analysis by FISH. Subsequent mutation analysis in this patient revealed L387 M mutation in the ABL domain of BCR/ABL oncogene. This patient is responding to an increased dose of imatinib.

**Figure 1:**
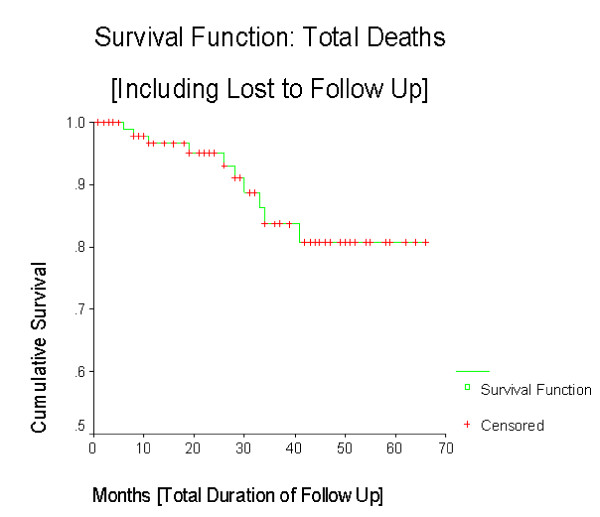
**Kaplan-Meier of cumulative survival of all patients**. This figure shows the cumulative survival of all patients enrolled in GIPAP.

**Figure 2:**
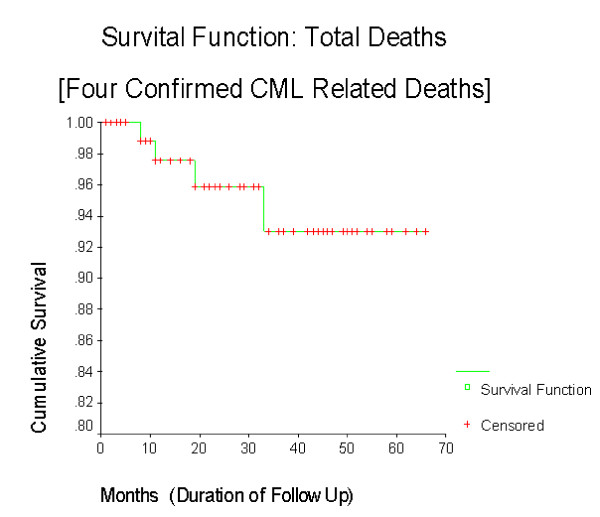
**Kaplan-Meier of cumulative survival of patients excluding those lost to follow-up**. The cumulative survival of patients, excluding those lost to follow-up, enrolled in GIPAP is shown.

**Figure 3:**
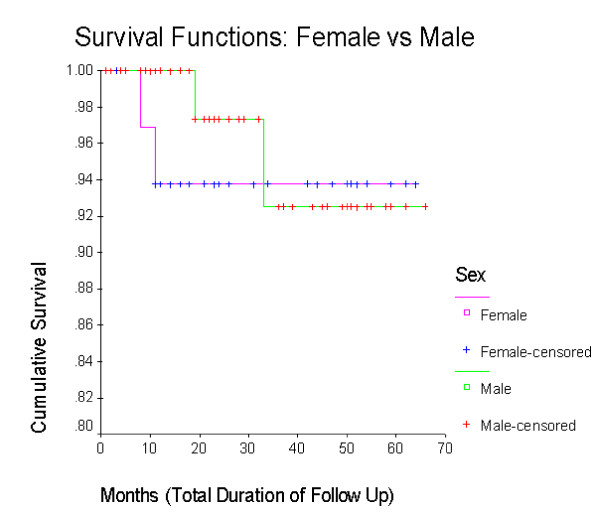
**Kaplan-Meier of survival based on gender**. The survival of patients, based on gender is shown.

**Figure 4:**
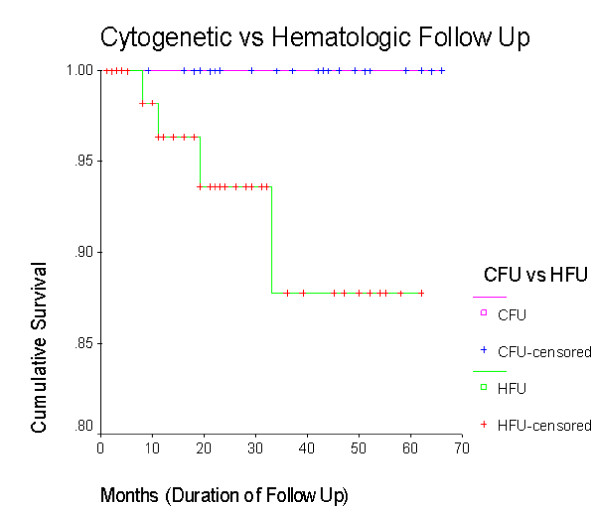
**Kaplan-Meier of survival based on cytogenetic or hematologic follow-up**. This figure demonstrates that the survival of patients who were able to obtain cytogenetic follow-up was superior to that of patients who only obtained hematologic follow-up.

The cumulative survival (including 2 deaths and 8 lost to follow up) is more than 80% (figure 1) with a mean follow up period of 27 months. The overall Survival function (with 4 confirmed CML deaths excluding lost to follow up) is 92% (figure 2) without any difference in mortality between sexes (figure 3). The cumulative survival approaches 100% in patients who underwent cytogenetic analysis (figure 4). Of the 103 CML patients on imatinib, about 90% of the patients did not progress to the accelerated phase or blast crisis with a median follow up of 24 months, and more than 90% of them are still alive.

We also looked at differences between characteristics of patients who were analyzed for both cytogenetic and hematologic responses compared to those who were analyzed for only hematologic response. All the patients who died or lost to follow up were from the group who only had hematologic analysis performed, and had not repeated cytogenetic analysis. Patients with cytogenetic analysis had a longer follow up. The cumulative survival approaches 100% in the group who had repeated cytogenetic analysis (figure 4).

## Discussion

The prevalence of CML is more common in the 31-50y age group in our study than in most demographic studies. The demographic characteristics between patients with or without cytogenetic analysis were not significantly different. Most of the patients were from Kathmandu and Lalitpur districts. Patients who had cytogenetic analysis repeated had a longer follow up period compared to the patients who had not. The increased number of deaths seen in patients who had not repeated cytogenetic analysis might be related to not being able to assess optimum responses to therapy.

Imatinib is currently the standard frontline therapy for treatment of CML. Druker and colleagues' five year follow-up of chronic phase CML patients treated initially with imatinib showed that the cumulative best rates for complete cytogenetic responses and complete hematologic response were 87% and 98% respectively[11]. An estimated 7% of the patients progressed to accelerated or blast phase. The overall survival rate was 89%, with more patients dying from causes unrelated to CML than their disease. Review of the International Randomized Study of Interferon vs STI571 (IRIS) data suggests that 63% of patients were successfully treated by imatinib at five years, while 37% of patients were no longer receiving the drug. In our study at Patan, 89.7% of our patients had major cytogenetic response (BCR-ABL1/Ph chromosome 0-34%), 6.9% of our patients had minor cytogenetic responses (BCR-ABL1/Ph chromosome 35-64%), and 3.4% of our patients had minimal cytogenetic response (BCR-ABL1/Ph chromosome 65-94%). After a mean follow up of 27 months (median follow up 24 months), CML had not progressed to the accelerated phase or blast crisis in 90% of the patients and more than 90% of the patients are still alive. Our patients' responses to imatinib were similar to those reported in IRIS by Druker and colleagues.

Importantly, our study establishes that imatinib is safe to use in a developing county. Myelosuppression was the most common side effect, and this was easily monitored in our setting with complete blood counts. Our monitoring allowed for appropriate dose adjustments.

Quite often patients on imatinib develop resistance, and the identification of resistance should trigger mutational analysis of BCR-ABL1 kinase domain. Reviews on managing imatinib resistance have been published[18-22]. A few of our patients treated with imatinib relapsed, reflecting the emergence of resistance to imatinib. Although there are multiple mechanisms involved in the emergence of resistance, usually resistance develops because of a mutation in the adenosine triphosphate-binding pocket of the BCR-ABL1 oncoprotein, which makes imatinib binding impossible. Molecular studies of resistant CML cells have been performed and have identified mutations that mediate this effect[23-27]. Several potent inhibitors of imatinib-resistant BCR-ABL1 have been identified and approved for clinical use[28-32]. Two of our patients developed secondary hematologic failure. Subsequent mutation analyses revealed mutations in ABL domain of BCR/ABL oncogene. The patient who had the L387 M mutation responded to dose escalation of Imatinib to 600 mg/day from 400 mg/day[33-36]. Another patient who had F359V mutation did not show response even with dose escalation to 800 mg/day. He was switched to second generation tyrosine kinase inhibitor (TKI) nilotinib but the response was not satisfactory. Failure to achieve major cytogenetic response by 12 months usually defines inadequate response[37]. He was then switched to dasatinib (another second generation TKI) and the response was remarkable. He went into hematological remission within few weeks. Dasatinib seems to be more effective in F359V mutation.

The main problem in our setting is the inability of our patients to obtain regular cytogenetic and molecular analyses as recommended by the standard guidelines in the management of Ph chromosome/BCR-ABL1 positive CML. The high cost of cytogenetic or molecular analysis and lack of availability of diagnostic centers in Nepal where these tests can be easily performed are prohibitive. Quantitative cytogenetic analysis by FISH costs about 7,000 Nepalese rupees (NRs) (US $90) and quantitative molecular analysis of BCR-ABL1/ABL ratio by RT-PCR costs about NRs. 10,000 (US $130). These costs are so high because the test is not done in Nepal; the laboratories only collect the samples and dispatch them to diagnostic facilities in India. Patients are often called back for repeat sample collections because the samples they have collected are lost somewhere in the chain of transport. Only about one-third of the total patients repeated cytogenetic analysis. The cumulative survival is significantly better in this group. Cytogenetic analysis allows detection of inadequate responses or resistance to therapy at an earlier stage of the disease and allows for adjustments in the management including choosing appropriate TKIs or referral for stem cell transplantation. Most of our patients could not afford a second test. So, it is very important that the concerned authorities seriously consider establishing an advanced medical laboratory at an affordable price in our own country.

Despite the fact that our patients hailed from different parts of Nepal, most patients managed to come for follow up regularly with usual hematological and biochemical tests and the compliance with the study medication was good. Interestingly, our patients also had remarkable cytogenetic and hematologic response rates consistent with other well controlled studies done elsewhere. GIPAP has enabled many patients to receive standard of care treatment that would otherwise be prohibitive due to cost in Nepal.

## Conclusions

Our study demonstrates that imatinib is safe to use in a developing country. Furthermore, we demonstrate that imatinib is very effective and induced long lasting responses in a high proportion of patients with Ph chromosome/BCR-ABL1 positive CML. Imatinib is well tolerated by our patients. The lack of cytogenetic analysis in the majority of our patients hindered our ability to detect inadequate responses to Imatinib and adjust therapy appropriately.

## List of abbreviations

CML: Chronic myeloid leukemia; GIPAP: Gleevec International Patient Assistance Program; Ph: Philadelphia; WBC: White blood cell; FISH: Fluorescent in situ hybridization; IRIS: International Randomized Study of Interferon vs STI571; TKI: Tyrosine kinase inhibitor; NRs: Nepalese rupees; CFU: cytogenetic follow-up; HFU: hematologic follow-up; HR: hematologic response; CR: cytogenetic response; CHR: complete hematologic response

## Competing interests

The authors declare that they have no competing interests.

## Authors' contributions

GK was responsible for the acquisition of data. All authors except AK were involved in the care of the patients enrolled in GIPAP. All authors have contributed to the drafting and review of the manuscript. All authors have given final approval for publication of this manuscript.

## Pre-publication history

The pre-publication history for this paper can be accessed here:

http://www.biomedcentral.com/1471-2326/10/8/prepub
